# Utilization of “prevention of mother-to-child transmission” of HIV services by adolescent and young mothers in Mulago Hospital, Uganda

**DOI:** 10.1186/s12879-018-3480-3

**Published:** 2018-11-14

**Authors:** Mariama Mustapha, Victor Musiime, Sabrina Bakeera-Kitaka, Joseph Rujumba, Nicolette Nabukeera-Barungi

**Affiliations:** 1 Department of Paediatrics and Child Health, School of Medicine, Makerere University College of Health Sciences, Kampala, Uganda; 2 Ministry of Health and Sanitation, Freetown, Sierra Leone; 3 Joint Clinical Research Centre, Kampala, Uganda

**Keywords:** Adolescents, HIV, PMTCT, Utilization, Uganda

## Abstract

**Background:**

Prevention of mother to child transmission (PMTCT) has lowered the incidence of paediatric HIV globally. The risk of mother-to-child transmission of HIV (MTCT) remains high in Africa, where there is a high prevalence of pregnancy and poor health-seeking behaviour among young girls and women.

**Methods:**

In this cross-sectional, mixed-methods study, we evaluated the utilization of PMTCT services and associated factors among adolescent and young postpartum mothers aged 15 to 24 years at a public urban referral hospital in Uganda. Both HIV-positive and HIV-negative participants were recruited. Utilization of PMTCT services was defined as use of the PMTCT cascade of services including ever testing for HIV, receiving HIV test results; If tested negative, subsequent retesting up to 14 weeks; If tested positive, Antiretroviral drugs (ARVs) for the mother, ARVs and septrin prophylaxis for infant, safe delivery, safer infant feeding, early infant diagnosis within 6 weeks, and linkage to treatment and care. Optimal utilization of PMTCT was defined as being up to date with utilization of PMTCT services for reported HIV status at the time of being interviewed. The overall proportion of participants who optimally utilized PMTCT services was determined using descriptive statistics. Qualitative data was analyzed manually using the content thematic approach.

**Results:**

Of the 418 participants, 65 (15.5%) were HIV positive. Overall, only 126 of 418 participants (30.1%) had optimally utilized PMTCT services. However, utilization of PMTCT services was better among HIV positive mothers, with 83% (54/65) having utilized the services optimally, compared to only 20% (72/353) of the HIV negative mothers (OR 18.2 (95% CI; 9.0–36.7)). The benefits of knowing ones HIV status, health of the unborn child, and counseling and support from health workers and peers, were the major factors motivating adolescent and young mothers to utilize PMTCT services, while stigma, financial constraints, non-disclosure, and lack of partner and family support were key demotivating factors.

**Conclusion:**

Utilization of PMTCT services by these adolescent and young mothers was suboptimal. Special consideration should be given to adolescents and young women in the design of elimination of mother to child transmission (EMTCT) programs, to improve the utilization of PMTCT services.

## Background

In 2016, there were 1.8 million new HIV infections, with a total of 36.7 million people living with HIV worldwide [1]. In high-prevalence settings, young women remain at unacceptably high risk of HIV infection [1]. In eastern Africa, young women (aged 15–24 years) accounted for 26% of new HIV infections in 2016 despite making up just 10% of the population [1].

Adolescents and young people remain extremely vulnerable to acquiring HIV infection, especially girls who live in settings with a generalized HIV epidemic or who are members of populations at high risk for HIV acquisition or transmission [2]. Although early diagnosis and treatment reduce HIV progression and prevent transmission, adolescents are less likely than adults to be tested, access care, remain in care and achieve viral suppression [2]. The Ministry of Health conducted an assessment of adolescent HIV and sexual and reproductive health care and treatment services at 30 health facilities in Uganda. This study found that pregnant adolescents (10–19 years) had less coverage of PMTCT services compared to mothers aged 20 years and above [3]. This study also found that 90% of pregnant adolescents attending antenatal care (ANC) took an HIV test but only 94% of those positive received ARVs [3]. This was in contrast with the adult mothers of whom 99% received ARVs for PMTCT [3].

Pregnant adolescent and young mothers have unique challenges that would hinder them from accessing HIV care [4]. For pregnant adolescents; obtaining access to relevant services, such as prenatal care, skilled attendants during birth, and PMTCT services, is more difficult [5]. Due to their young age, teenage mothers have to deal with disapproving health care providers. In addition, those living with HIV may face stigma and discrimination in health care settings [5].

PMTCT covers a package of interventions summarized as 4 prongs, which should be implemented simultaneously: Primary prevention of HIV infection among women of childbearing age, preventing unintended pregnancies among women living with HIV, preventing HIV transmission from a woman living with HIV to her infant using option B+ approach, and providing appropriate treatment, care and support to mothers living with HIV and their children and families [6]. The PMTCT cascade is a series of key stepwise activities that begins with all pregnant women and ends with the detection of a final HIV status in HIV-exposed infants [7]. The cascade comprises 18 months of care and includes attending antenatal care (ANC), HIV testing and counselling during ANC, receiving HIV test results; If tested negative, subsequent retesting; If tested positive, antiretroviral drugs (ARVs) for the mother, ARVs and septrin prophylaxis for the infant(s), safe delivery, safer infant feeding, early infant diagnosis within 6 weeks, second DNA PCR test done 6 weeks after cessation of the breastfeeding, serology at 18 months if DNA PCR negative, and linkage to treatment and care [7]. Optimal utilization of the PMTCT cascade of services has not been previously described in the literature.

Initiation and completion of the PMTCT cascade is important for elimination of paediatric HIV [8]. Adolescents have poor health seeking behaviour if services are not adolescent friendly, and few facilities in Uganda were found to be adolescent friendly in a 2007 study [9]. Since over 90% of new HIV infections among infants and young children occur through MTCT, it is certain that PMTCT remains the top priority. A focus on women is a key strategy to preventing/reducing HIV infection among children [10]. PMTCT programs not only reduce transmission of HIV, but if well implemented as part of a full continuum of care, they can result in HIV-free survival, meaning that infants are protected from other causes of death as well.

Thirty percent of Uganda’s 35 million people is comprised of adolescents and young people aged 10–24 years [11], with a birth rate of 140 per 1000 among adolescents by age 19 years [12]. This group may need PMTCT services and messages that are different from those which target older women. Although adolescent and young mothers have been noted to have poor utilization of health services (6), there is limited data on the utilization of PMTCT of HIV services by adolescent and young mothers in Uganda. Identifying the gaps in the utilization of PMTCT services and the associated factors will contribute to the development of practical strategies in policy and practice to improve utilization of PMTCT services by these young mothers, and hence achieve elimination of paediatric HIV infection.

We described the utilization of PMTCT of HIV services and the associated factors among adolescent and young mothers at a public urban referral hospital in Uganda.

## Methods

### Study design

This was cross-sectional and mixed methods study which employed both quantitative and qualitative methods.

### Study site

This study was conducted at the immunization, postnatal and family planning clinics at Mulago National Referral Hospital, located in Kampala, the capital city of Uganda. Mulago Hospital serves about 115, 000 inpatients (including 30, 000 children) per year. These clinics are located in the same building in upper Mulago Hospital, work hand-in-hand, and run Mondays through Fridays. The median number of postpartum mothers seen in the clinics daily is 80. Of these 80 women, 5–10 (6–13%) are adolescent and young women. Routine HIV testing is performed by a trained counsellor, and those who test positive are linked either to Baylor-Uganda Clinical Centre of Excellence or to the PMTCT follow-up clinic, both within a 200 m radius.

### Quantitative component

#### Study population

The study population for the quantitative component of the study was all postpartum mothers (HIV positive and negative) aged 15–24 years attending the clinics who: provided written informed consent, and had a child or children aged 9 months and below. Age 9 months and below was chosen for the infants’ age in order to include mothers whose pregnancy was therefore recent, and hence minimize recall bias.

#### Sample size estimation

Utilization of PMTCT services is use of the PMTCT cascade of services including testing for HIV, receiving HIV test results; If tested negative, subsequent retesting up to 14 weeks; If tested positive, Antiretroviral drugs (ARVs) for the mother, ARVs and septrin prophylaxis for infant, safe delivery, safer infant feeding, early infant diagnosis within 6 weeks, and linkage to treatment and care. Optimal utilization of PMTCT is being up to date with utilization of PMTCT services for reported HIV status at the time of being interviewed. Testing for HIV is a crucial first step in utilization of the PMTCT cascade of services for both HIV negative and HIV positive women. Using an HIV testing rate of 61.7 [13], a sample size of 363 participants, provided 90% power to assess the optimal utilization of PMTCT services in this population allowing for a 10% data loss through data collection constraints and errors.

#### Quantitative data collection

Two interviewers (nurses) received a two-day training by the researcher on the questionnaire, data collection procedures and sampling methods. They were available at the clinics on all clinic days and were supervised by the researcher. A pre-tested structured questionnaire was used to obtain information on the participants’ socio-demographic characteristics, HIV/AIDS and PMTCT-related knowledge, PMTCT utilization, and factors influencing utilization of PMTCT services. The nurses administered the questionnaire using face-to-face exit interviews from March–June 2015. All postpartum mothers attending the clinics were invited to participate. A woman was eligible if she was 15–24 years old, had a child aged 9 months and below, and consented for interview.

#### Quantitative data management and analysis

Completed questionnaires were scrutinized by the researcher on the spot for immediate correction of erroneous entry, and feedback given to the research assistants. After data collection, all questionnaires were stored in a safe that was accessible only to the researcher. Data was cleaned, coded, and double-entered into Epidata version 3.1. Range, consistency and validity checks were built in to minimize errors. A back up copy of the data was kept on an external hard drive. Data was exported and analyzed using STATA version 12.0 (STATA Corporation, Houston, Texas). A descriptive analysis was done so as to depict the baseline characteristics of the study population. The distribution of the participants’ characteristics was presented as frequencies with respective proportions.

The primary outcome of the study was optimal utilization of PMTCT services as defined by being up to date with utilization of PMTCT services. This meant that at that particular point in time when the mother was interviewed during the study, she was where she was supposed to be at the PMTCT cascade for her reported HIV status.

A logistic regression model was used to assess the association of selected variables with the overall optimal utilization of PMTCT services. Bivariate factors with a *p*-value < 0.2 were considered for multivariate analysis. The distribution of each selected variable was compared between those who optimally utilized PMTCT services and those who did not. In all analyses, a p-value of < 0.05 was taken as statistically significant.

### Qualitative component

#### Study population

To complement the quantitative findings, interviews were held with purposively selected health workers involved in the provision of PMTCT services at Mulago Hospital, and purposively selected adolescent and young mothers (HIV positive and negative) with either good or poor utilization of PMTCT services.

### Qualitative data collection

#### In-depth interviews (IDI)

During the study, after administration of the questionnaire, adolescent and young mothers for participation in the qualitative component were identified. IDIs were conducted with mothers who provided consent. Twenty adolescent and young mothers (10 HIV positive and 10 HIV negative) were interviewed. After the 20 interviews, no further interviews were conducted since no new information was being generated. IDIs were aimed at obtaining an in-depth understanding of the facilitators and barriers to utilization of PMTCT services by adolescent and young mothers. In-depth interviews were chosen to allow free and confidential interaction between researchers and adolescent mothers. The interviews took the form of informal conversations and were conducted in the local language, *Luganda*, which was the language preferred by the mothers. The interviews were conducted by a research assistant with experience in qualitative studies involving adolescents and young people. A pre-tested flexible interview guide with probes was used to explore the ideas and experiences of the participants regarding HIV testing among adolescent and young mothers, and the factors that influence the utilization of PMTCT services, and suggestions for improving utilization of PMTCT services by this group. The interviews were audio recorded (except for 3 mothers who were not comfortable with being recorded), transcribed and translated by the research assistant. Each interview took an average of 35 min.

#### Key-informant interviews (KII)

KIIs were held on appointment with selected participants at the health facility during the study. Nine key informant interviews were conducted, and these included 2 nurses, 2 counsellors, 3 peer educators, and 2 doctors who were both PMTCT coordinators. Key informants were selected based on their involvement in the provision of PMTCT services and were intended to contribute to a better understanding of adolescent and young mothers’ experiences with PMTCT services. The interviews were guided by a pre-tested flexible interview guide with probes to identify the characteristics of the health workers, their role in providing PMTCT services, their views on the motivators and barriers to the utilization of PMTCT services by adolescent and young mothers, and their suggestions for improving utilization of PMTCT services by this group. Interviews were conducted in English, audio recorded, and transcribed by the research assistant experienced in conducting qualitative research. Each interview lasted about 35 min.

#### Qualitative data management and analysis

Qualitative data was analysed manually using the content thematic approach [14]. Data analysis was done by the first author in collaboration with the fourth author (an experienced qualitative researcher). This involved reading scripts several times, identifying themes and sub-themes, and grouping data according to these themes for interpretation. The main study themes were; motivators and barriers to utilization of PMTCT services. All co-authors were involved in discussions of study themes, sub-themes and interpretation of findings. This process facilitated researcher triangulation [15] to attain a broader understanding of adolescent and young mothers motivators and barriers to utilization of PMTCT services. Direct quotations from study participants reflecting motivators and barriers to use of PMTCT services were identified and used in the presentation of the study findings.

## Ethical considerations

Ethical approval for the study was obtained from the Makerere University College of Health Sciences School of Medicine Research Ethics Committee. Written informed consent was obtained from eligible mothers and health workers who agreed to participate in the study. Study participants below 18 years of age independently provided informed consent as emancipated minors, due to them having children, under the Uganda National Council for Science and Technology (UNCST) National Guidelines for Research [16]. The interviewers were instructed on how to comply with strict confidentiality practices for all clients both during and after data collection. For purposes of confidentiality only study specific serial numbers were used in data entry and analysis and the data was only accessible to the research team.

## Results

### Characteristics of the study population

We screened 453 adolescent and young postpartum mothers attending the Mulago Hospital immunization, postnatal and family planning clinics during the study period (March to June 2015). Of these, 418 were enrolled and interviewed. Out of 35 mothers not enrolled, 25 declined consent for participation, and 10 had children older than 9 months of age.

The median age of the participants was 22 years (IQR 15–24 years). Of the 418 participants, 65 (15.5%) were HIV positive, and 353 (84.5%) were HIV negative.

The majority of the 418 participants were married (76.6%), had a secondary education (62.0%), not employed (66.3%), had a monthly household income between 50,000 and 200,000 Ugandan shillings (15–59 USD) (70.1%), used public transport to get to the facility (71.0%), and attended antenatal care at least once during their most recent pregnancy (98.6%). Only 81 mothers (19.7%) had attended ANC four or more times as shown in Table 1.

**Table 1:** Characteristics of 418 mothers attending immunization, postnatal, and family planning clinics of Mulago Hospital, Kampala in 2015

Variable	Distribution of participants	
	Number	Percentage
Age categories (years)		
15–19	76	18.2
20–24	342	81.8
Marital status		
Single	67	16.0
Married	320	76.6
Co-habiting	23	5.5
Separated	8	1.9
Education		
Primary	107	25.6
Secondary	259	62.0
Tertiary	47	11.2
Never	5	1.2
Employment status		
Employed	141	33.7
Not employed	277	66.3
Religion		
Christian	335	80.1
Moslem	83	19.9
Combined Monthly income^a^		
< 50,000	20	4.8
50,000-200,000	293	70.1
200,001-500,000	100	23.9
> 500,000	5	1.2
Travel means to the clinic		
Walk	114	27.3
Public transport	297	71.0
Private transport	7	1.7
Attend ANC for most recent pregnancy		
No	6	1.4
Yes	412	98.6
Number of ANC attendances		
< 4	331	80.3
≥ 4	81	19.7
Parity		
One	294	70.3
Two	101	24.2
Three	20	4.8
Four	3	0.7
Reported HIV status^b^		
Positive	65	15.5
Negative	353	84.5

^a^Combined monthly income in Ugandan Shillings

^b^Reported HIV Status: As was reported by the mothers

### Utilization of PMTCT services

Of the 418 participants, all of them (100%) had ever tested for HIV and received their test results. Of these, 400 (95.7%) tested during their most recent pregnancy, while 18 (4.3%) tested prior to their most recent pregnancy. Of the 400 mothers who tested for HIV during their most recent pregnancy, 395 (98.7%) tested in ANC, while 5 (1.3%) tested out of ANC.

Of the 418 participants, 65 (15.5%) reported that they tested HIV positive, and 353 (84.5%) reported that they tested HIV negative. The participants’ self-reported HIV status was verified from clinic records.

Of the 353 participants who tested HIV negative, 154 (43.6%) did not retest after receiving their first test results and hence were not retained in the HIV negative PMTCT cascade. Of the 199 who retested for HIV, 97 (49%) retested during the third trimester of their pregnancy, and 30 (15%) retested during labour/delivery, but were not up to date with retesting for HIV, resulting in delays in retesting for HIV after the first negative test. Of the 199 who retested for HIV, only 72 (36%) were up to date with retesting for HIV as was recommended.

Of the 65 mothers who tested HIV positive, 64 (98.5%) were enrolled in HIV care and used PMTCT ARVs. One mother was not enrolled in care because she declined enrolment and was not retained in the PMTCT cascade. Three of the 64 mothers who were enrolled in HIV care reported that their babies tested HIV positive by DNA PCR done at 6 weeks of age, giving an HIV transmission rate of 4.7%. The reported HIV status of the babies was verified from clinic records. The characteristics of the mothers of these 3 babies were similar to those of the other mothers whose babies were reportedly HIV negative. Among the infants of the 64 mothers who were enrolled in care, one was not started on ARV prophylaxis at birth for unknown reasons. There were delays in the utilization of the PMTCT cascade for four mothers whose infants were not started on septrin prophylaxis at 6 weeks, and 8 infants whose DNA PCR was not done at 6 weeks of age.

**Fig. 1:**
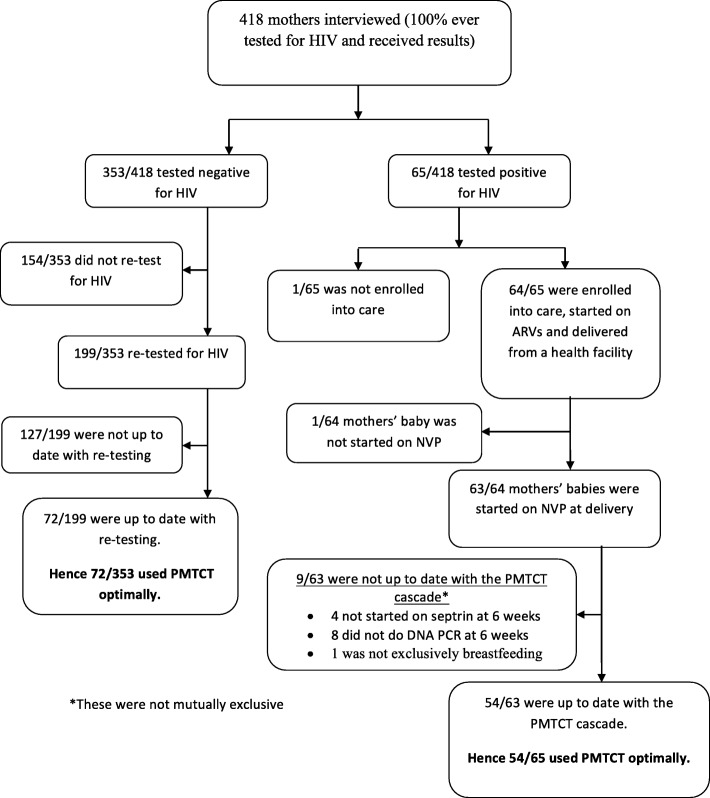
Utilization of PMTCT cascade for 418 adolescent and young mothers attending Mulago Hospital

The details of the utilization of the early PMTCT cascade for 418 adolescent and young mothers attending Mulago Hospital are shown in Fig. 1.

### Optimal utilization of PMTCT services

Utilization of PMTCT services was higher among HIV positive mothers, with 54 of the 65 (83.1%) HIV positive mothers having utilized the services optimally, compared to only 72 of the 353 (20.4%) HIV negative mothers (OR 18.2 (95% CI: 9.0–36.7)).

A higher proportion of mothers aged 20–24 years (32.5%), regardless of HIV status, had optimally utilized the services compared to only 19.7% of mothers aged 15–19 years (OR 1.9 (95% CI: 1.1–3.6)).

### Factors associated with optimal utilization of PMTCT services

Age and reported HIV status were significantly associated with optimal utilization of PMTCT services at bivariate analysis. Compared to women in the age group 15–19 years, women in the age group 20–24 years were more likely to optimally utilize PMTCT services (OR 1.9 (95% CI: 1.1–3.6); *p*-value = 0.031).

The factors at bivariate analysis with a p-value < 0.2 which were considered for multivariate analysis, included: age, reported HIV status, and attendance of ANC. Of these 3 variables, only reported HIV status was significantly associated with optimal utilization of PMTCT services at multivariate analysis, (OR 18.2 (95% CI: 9.0–36.7)), as shown in Table 2.

**Table 2:** Adjusted analysis of factors independently associated with optimal utilization of PMTCT services by adolescent and young mothers attending Mulago Hospital

Variable	Adjusted odds ratio (95% CI)	*P* value
Age category		
15–19	1.0	
20–24	1.8 (0.9–3.5)	0.110
Reported HIV status		
Negative	1.0	
Positive	18.2 (9.0–36.7)	0.001
Attended ANC		
No	1.0	
Yes	0.3 (0.04–2.6)	0.299

### Motivators for utilization of PMTCT services

The benefits of knowing HIV status (90.2%) was the commonest reported motivating factor for utilizing PMTCT services. Other reported motivating factors included health of unborn child (81.3%), responsibility to prevent spread of HIV (73.2%), desire to know ones’ status (72.97%), counselling by health care staff (69.1%), perception of need (61.7%), and peer and family support (34.9%), as shown in Table 3.

**Table 3:** Perceived motivators of utilization of PMTCT services by adolescent and young mothers attending Mulago Hospital

Motivating Factor	Percentage^a^ (*N* = 418)
Benefits of knowing HIV status	90.19
Health of unborn child	81.34
Responsibility to prevent spread of HIV	73.21
Desire to know ones status	72.97
Counselling by health care staff	69.14
Perception of need	61.72
Peer and family support	34.93

^a^Percentages add up to > 100% given non-mutually exclusive answer count

### Barriers to utilization of PMTCT services

**Fig. 2:**
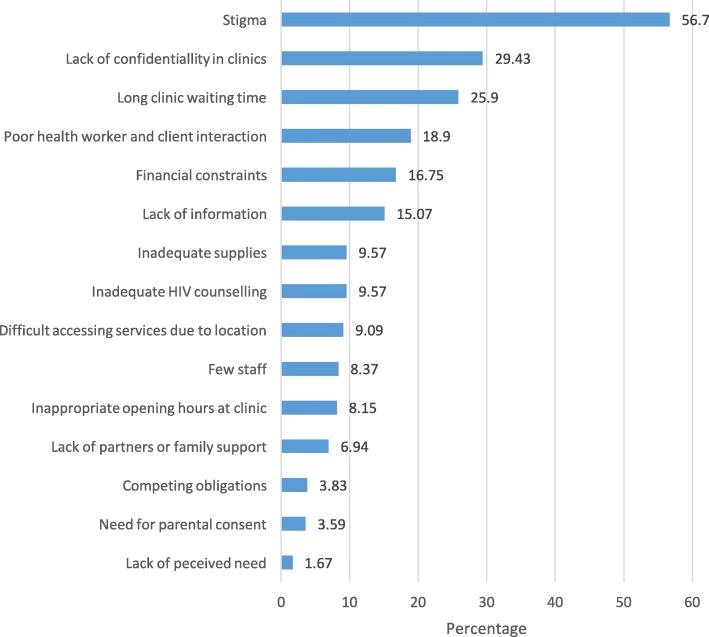
Perceived barriers to utilization of PMTCT services by adolescent and young mothers attending Mulago Hospital

Stigma (56.7%) was the commonest reported barrier to adolescent and young mothers’ utilization of PMTCT services. Other commonly reported barriers included lack of confidentiality in the clinics (29.4%), long waiting times at the clinics (25.9%), poor health worker and client interaction (18.9%), financial constraints (16.7%), and limited information (15.1%), as shown in Fig. 2.

### Qualitative study findings

Of the 20 mothers involved in the IDIs, 6 were aged 15–19 years (2 HIV positive and 4 HIV negative), while 14 were aged 20–24 years (8 HIV positive and 6 HIV negative). A total of 9 key informants were interviewed. All of the key informants were females (only females were found to be involved in the provision of the PMTCT services), 4 out of 9 were aged 30 years or less. Three of the KIs had been involved in providing PMTCT services for less than 5 years, four had been involved in providing PMTCT services between 5 and 10 years, and two had been involved in providing PMTCT services for more than 10 years.

### HIV testing knowledge and practices

Data from both IDIs and KIIs showed that most mothers had heard about HIV testing prior to their coming to Mulago Hospital. Most mothers had learnt about HIV testing from the media, friends, and from ANC for previous pregnancies.

HIV testing as part of ANC was not a surprise for the adolescent and young mothers, yet many found it a difficult step to take. Most believed that HIV testing was only necessary when a woman is sick or pregnant as one participant noted:

*It is not easy because some young mothers say they cannot look for trouble by coming to test for HIV. They wait to get a reason for testing such as pregnancy or sickness and then they will be forced to test because during antenatal it is a must to test… (Single, 23 years, HIV positive).*

Consistent with quantitative findings, qualitative interviews revealed that all of the study participants had tested for HIV, mainly during pregnancy.

Generally, the HIV negative participants thought retesting for HIV after a first negative test was easy, although not many go back to retest. The HIV positive participants however thought that retesting was not easy for HIV negative women, for fear of the results turning positive, and the fact that they saw it as unnecessary. This sentiment was also shared by the health workers, who thought that the women see no need to retest after testing HIV negative the first time.

However, many narratives of HIV positive mothers indicated that they had at onetime tested HIV negative, and later tested HIV positive.

*During the time I used to go for antenatal, I was tested and on all occasions my results showed that I was HIV negative... However, when I was tested again after delivery I was found to be positive... (Separated, 21 years, HIV positive).*

### Motivators for utilization of PMTCT services

In the IDIs, the participants revealed that services were better for HIV positive women as compared to HIV negative ones which could influence their utilization of PMTCT services. HIV positive women had more services available to them such as income generation activities and peer support.

*The care and attention that health workers give us is very encouraging and can motivate other mothers to also utilize the services... (Married, 23 years, HIV positive).*

This was further highlighted by the health workers in the KI interviews.

Consistent with quantitative findings, most participants in the IDIs mentioned the benefits of knowing their HIV status, such as getting access to free treatment and services, as a strong motivator for utilization of PMTCT services. This view was also shared by the health workers.

The desire to protect their unborn children from acquiring HIV infection was another dominant motivator for these women.

*Most young mothers who utilize PMTCT services are driven by the desire to protect their children from infection. Sometimes a young mother could have given up on themselves but when they teach them that their babies can be HIV negative, they begin to use the service for the sake of the baby.. (Separated, 21 years, HIV positive).*

In-depth and KI interviews further revealed that counselling and support by health care staff and peer educators were strong motivators for mothers to utilize PMTCT services.

*The health workers and peer educators are actually good because they take the initiative and call you in case you default on your refills for the baby. They call and remind you to come for your drugs or send someone to pick them for you in case you are unable to. (Cohabiting, 22 years, HIV positive).*

*At the PMTCT clinic the peer educators who are also HIV positive share their testimonies with these mothers to encourage them and help them understand that one can live positively and live healthy. Most of the peer educators have been beneficiaries of PMTCT and their children are negative. This encourages mothers to adhere to treatment as well as to continue coming for the services.. (Peer educator).*

The personal experiences of these mothers further highlighted the different factors that motivated them to utilize PMTCT services. Such motivators were; a longing to learn about HIV, fear of death, and desire to protect the baby from getting infected with HIV.

### Barriers to utilization of PMTCT services

Consistent with quantitative findings, individual factors, health services related factors, as well as societal factors came up as barriers to utilization of PMTCT services in both the in-depth interviews and the KI interviews.

#### Stigma

Stigma emerged as a major barrier to the participants’ utilization of PMTCT services as noted by one mother:

*Stigma is a major issue. Young mothers fear that their results will be shared or discovered by other people especially if they test positive.. (Cohabiting, 23 years, HIV negative).*

Similarly, key informants stressed the role of stigma as a barrier to utilization of PMTCT services by was noted by one key informant:

*Stigma is the biggest challenge adolescent mother’s face. They first of all stigmatize themselves and then the stigma that comes from the community. Young mothers think that when one is tested positive, they are going to die and they lose hope in themselves... (Peer educator).*

#### Individual level barriers

At the individual level, financial constraints, competing obligations, non-disclosure, lack of symptoms, lack of partner and family support, lack of information, and fear of drugs stood out as factors that prevent adolescent and young mothers from utilizing PMTCT services.

*I am not ready to disclose to my parents yet...It was impossible for me to practice exclusive breast feeding because each time the baby cried, my grandmother and the people around would tell me to give him food and then breastfeed him.. (Married, 23 years, HIV positive).*

Fear to swallow drugs for a long period of time was another factor hindering adolescent and young mothers from utilizing PMTCT services.

*Young mothers fear to swallow drugs. When they are told that they have to take the drugs every day for their life time, they become tired even before starting... (Peer educator).*

#### Health services barriers

At the health services level, negative attitude of health workers stood out as a barrier to utilization of PMTCT services.

*There is a particular health worker who abuses young girls a lot for being pregnant. Some mothers used to run away before being attended to... (Cohabiting, 20 years, HIV negative).*

*Some nurses are very harsh to mothers especially when they are tired. They become very rude, and this can discourage the mothers from coming back to the clinics .. (Single, 23 years, HIV positive).*

Long waiting time at the clinic, long distance to the health facility, and lack of adolescent specific clinics were also mentioned as barriers to utilization of PMTCT services. Waiting time was related to the high number of clients compared to health workers.

*The clinic receives very many mothers, and this increases their waiting time...Considering the fact that they are either pregnant or nursing babies, they don’t like waiting for a long time (Counsellor).*

*When mothers stay far from the hospital and need to pay for transport, they could be discouraged especially if they are not working and have no one giving them financial support... (Single, 21 years old, HIV positive)*

## Discussion

The key finding in this study is that the proportion of adolescent and young mothers who had optimally utilized PMTCT services was low. Optimal utilization of PMTCT services was defined as being up to date with utilization of the PMTCT cascade of services for reported HIV status at the time the interview was conducted. There is limited information on optimal utilization of the PMTCT cascade of services as defined in this study, as most studies only looked at one step or the other of the cascade, and not the entire cascade as a whole [17, 18]. Optimal utilization of PMTCT services was significantly associated with reported positive HIV status. One explanation could be that programs in the hospital designed to improve PMTCT uptake have been focussing on HIV positive mothers. These programs use highly motivated peer educators to counsel and follow up the HIV positive mothers, and also have income generating and psychosocial support activities for them further encouraging their utilization of the services. Overall, the support for HIV negative adolescent and young women seem to end after communicating negative results. This emphasizes the need for sustained HIV prevention efforts to scale up primary prevention among young HIV negative women. Another explanation could be that the HIV positive mothers included in this study formed the population of HIV positive women who came back for clinic services, and so are better at utilizing PMTCT services compared to the population who do not come back. Hence there is a potential for overestimation of retention in the PMTCT cascade among this population of HIV positive mothers included in this study.

In this study, only 20% of HIV negative mothers had optimally utilized PMTCT services. As much as 43.6% of them did not retest for HIV after the first test, and of those who retested, more than 60% were not up to date with retesting for HIV. Whereas it is understandable for more support to be accorded to women who test HIV positive, the apparent inattention given to those who tested HIV negative the first time could explain their poor utilization of PMTCT services. It is however important to note that some mothers who test HIV negative the first time could test HIV positive later on in the pregnancy or after delivery. New HIV infections could remain undetected in previously HIV negative mothers due to their low rates of retesting, leading to undiagnosed maternal infection, and hence mother-to-child transmission of HIV [7]. This highlights the importance of retaining HIV-negative women in the PMTCT cascade.

This study found a high uptake of PMTCT services (98.5%) among HIV positive participants including enrolment into care, taking ARVs and delivering from a health facility. These findings closely align with the report by the Uganda’s Ministry of Health (MOH) that more than 90% of HIV positive pregnant women were enrolled in care, received ARVs for themselves and their babies, and exclusively breastfed their babies in Uganda in 2013 [19]. Other African studies have reported similar findings [20, 21]. Given the efficacy of Antiretroviral Therapy (ART) in HIV-infection control, and especially where adherence to treatment is good, high program uptake can translate into a significant reduction in the burden of HIV infections in the paediatric population. The MTCT rate of 4.7% found among the participants is lower than that reported previously [22], prior to introduction of the World Health Organization (WHO) option B- plus strategy in Uganda, but it still falls below the elimination target of < 2% [23].

This study highlights a number of issues useful for understanding factors influencing the utilization of PMTCT services. The study also documented potential areas for improving PMTCT interventions.

Adolescents had less utilization than young women, and this was significant at bivariate analysis. Although this significance fell out at multivariate analysis, adolescents have been noted to have less utilization of PMTCT services (4).

Health workers in this study recognized that psychosocial support and the engagement of peer educators encouraged adolescent and young mothers to utilize PMTCT services. Studies in Uganda have shown that adolescents experience a lot of psychosocial challenges especially when pregnant [4, 9], hence it is no surprise that continuous support and counselling of these mothers would increase their utilization of these services. Peer support should be maximized in all centres offering PMTCT services in order to improve utilization of these services.

This study further emphasizes the fact that the desire to protect the baby from becoming infected with HIV is a strong motivator for young mothers’ utilization of PMTCT services. This implies that focussing on the baby in prevention messages could improve utilization of PMTCT services in this vulnerable group.

This study has revealed multiple social, cultural, economic and physical barriers that might hinder the success of PMTCT interventions. Stigma was found to be a key demotivating factor for adolescent and young mothers’ utilization of PMTCT services. A study in Kenya found that stigma and health system factors were barriers to utilization of PMTCT services [21]. Stigma remains a major concern with women not wishing to have their HIV status disclosed. Many women are therefore reluctant to utilize PMTCT services for fear of being stigmatized and discriminated against, emphasizing the importance of addressing stigma as a barrier, if EMTCT is to be realized.

This study has several strengths: The study contributes to the literature by identifying some of the gaps in the utilization of PMTCT services which were not previously described. The study had both quantitative and qualitative aspects. Qualitative interviews involving use of probes and triangulation of data from different sources helped to improve the trustworthiness of the study findings. Furthermore, the inclusion of HIV positive and HIV negative mothers in the study provided an opportunity to uncover the unique experiences and support needs for each of the two groups of adolescent and young women.

The findings of this study should however be interpreted in view of the following limitations:

Recall bias cannot be excluded. However, analysis was limited to mothers whose infants were 9 months old or younger and whose pregnancy was therefore recent. The fact that quantitative interviews were conducted by nurses may have affected participants’ response to perceived barriers to utilization of PMTCT services. The study was conducted at the National Referral Hospital, a public hospital characterized by congestion and delays at the clinics. It is possible that women with higher incomes obtain care from private clinics and may thus be under-represented in this study. Optimal utilization of PMTCT services in this study was determined for the first 9 months of the recommended PMTCT cascade (in order to minimize recall bias), instead of the entire PMTCT cascade of 18 months. The relatively smaller number of HIV positive women included in the study (*N* = 65), and their unequal distribution (only 7 were adolescents aged 15–19 years) could have affected the results of this study. However, these HIV positive mothers provided very useful information with regards to the motivators and barriers of their utilization of PMTCT services.

## Conclusions

Optimal utilization of PMTCT services among adolescent and young mothers at 30% was low. However, optimal utilization of PMTCT services was better among the HIV positive women as majority (83%) of them had optimally utilized the services, compared to only 20% of the HIV negative mothers. The study further demonstrated losses and delays throughout the PMTCT cascade. Reported positive HIV status was significantly associated with optimal utilization of PMTCT services. The major factors motivating adolescent and young mothers to utilize PMTCT services included: the benefits of knowing ones’ HIV status, health of the unborn child, and counselling and support by health care staff. Stigma was a key demotivating factor for the adolescent and young mothers’ utilization of PMTCT services.

We recommend that special consideration should be given to adolescents and young women in the design of EMTCT programs, to improve the utilization of PMTCT services particularly among those found HIV negative at the first HIV test to support them to remain HIV negative. In addition, we recommend that stigma as a barrier to the utilization of PMTCT services should be addressed by PMTCT service providers and policy makers, and that counselling and health care support should emphasize on the benefits of PMTCT services since they motivate adolescent and young mothers to utilize the services.

## Abbreviations

AIDS: Acquired immunodeficiency syndrome
ANC: Antenatal care
ART: Antiretroviral Therapy
ARV: Antiretroviral drug
DNA PCR: Deoxyribonucleic acid polymerase chain reaction
EMTCT: Elimination of mother-to-child transmission (of HIV)
HIV: Human immunodeficiency virus
IDI: In-depth interviews
KII: Key-informant interviews
MOH: Ministry of Health
MTCT: Mother-to-child transmission (of HIV)
NVP: Nevirapine syrup
PMTCT: Prevention of mother-to-child transmission (of HIV)
UNCST: Uganda National Council for Science and Technology
WHO: World Health Organization
